# Structure and Properties of Metastable β Ti–25Nb–8Sn Alloy following Cold Rolling and Aging Treatments

**DOI:** 10.3390/ma17133062

**Published:** 2024-06-21

**Authors:** Hsueh-Chuan Hsu, Shih-Ching Wu, Zhong-Lin Jian, Wen-Fu Ho

**Affiliations:** 1 Department of Dental Technology and Materials Science, Central Taiwan University of Science and Technology, Taichung 40601, Taiwan; hchsu@ctust.edu.tw (H.-C.H.); scwu@ctust.edu.tw (S.-C.W.); 2 Department of Chemical and Materials Engineering, National University of Kaohsiung, Kaohsiung 811726, Taiwan

**Keywords:** titanium alloy, cold rolling, aging treatment, phase transformation, mechanical properties, corrosion properties

## Abstract

Metal implants require an elastic modulus close to cortical bone (<30 GPa) to avoid stress shielding and ensure adequate load-bearing strength. The metastable β-type Ti–25Nb–8Sn alloy has a low elastic modulus (52 GPa), but its yield strength (<500 MPa) needs enhancement. This study enhances Ti–25Nb–8Sn’s elastic admissible strain through cold rolling and aging heat treatments, investigating the microstructure’s impact on mechanical and corrosion properties. The results show that lower-temperature aging (<450 °C) leads to ω-phase precipitation, yielding a 300% increase in yield strength (>1900 MPa). However, this also increases the elastic modulus (~80 GPa), limiting the deformation ability. Higher-temperature aging (>500 °C) eliminates the ω phase, transforming it into α precipitates, resulting in a lower elastic modulus (~65 GPa) and improved deformation ability, with substantial yield strength (>1000 MPa). In summary, the optimal process conditions are determined as 90% cold rolling followed by aging treatment at 550 °C. Under these conditions, Ti–25Nb–8Sn achieves the most suitable yield strength (1207 MPa) and high corrosion resistance, retaining a relatively low elastic modulus (64.7 GPa) and high elastic admissible strain (1.93%). This positions it as an ideal material for biomedical implants.

## 1. Introduction

Titanium (Ti) alloys have garnered significant attention across various industries owing to their remarkable biocompatibility, high corrosion resistance, specific strength, and low elastic modulus [1]. Among these alloys, Ti–6Al–4V ELI stands out as a widely utilized material in biomedical implants [2]. However, its elastic modulus (~110 GPa) exceeds that of human bone (<20 GPa), leading to a phenomenon known as stress shielding [2]. This effect transfers the majority of stress to the implant, consequently inducing bone resorption and eventual implant failure [2]. Compounding this, concerns regarding the cytotoxicity of vanadium (V) ions and the potential link between aluminum (Al) ions and Alzheimer’s disease have prompted the exploration of alternative alloying elements such as niobium (Nb), tin (Sn), tantalum (Ta), zirconium (Zr), and molybdenum (Mo) in biomedical Ti alloys [3].

Metastable β-type Ti alloys undergo phase transformation, generating α″ phase upon cold rolling, thereby refining β-phase grains [4]. This refinement contributes to enhanced strength and reduced elastic modulus [5]. Previous studies have demonstrated the efficacy of cold rolling in enhancing mechanical properties. For instance, Fu et al. [6] observed significant improvements in tensile strength and elastic modulus in the Ti–15Nb–5Zr–4Sn–1Fe alloy subjected to varying degrees of cold rolling. Similarly, Yumak et al. [7] observed that the metastable β Ti alloy precipitated α phase after heat treatment (550 °C, 20 h), resulting in an increase in strength. However, this enhancement brought about by long-term heat treatment is often accompanied by a concomitant increase in elastic modulus [7,8]. Therefore, optimizing heat treatment conditions becomes imperative to achieve the desired mechanical properties.

Past research has extensively investigated the effects of cold rolling and subsequent heat treatments on various Ti alloys. For instance, Matsumoto et al. [9] conducted 89% cold rolling on Ti–35Nb–4Sn followed by solution treatment at 573 K. Their results indicated a weakening of the intensities of the XRD diffraction peaks of the α″ phase formed by rolling after solution treatment. Moreover, solution treatment induced a reverse transformation from α″ phase to β phase and precipitated the α phase, thereby strengthening the alloy. Similarly, Du et al. [10] highlighted the precipitation of the ω phase in the Ti–10Mo–6Zr–4Sn–3Nb alloy during aging, resulting in increased strength but also brittleness. Conversely, Vishnu et al. [11] conducted aging treatments on Ti–35Nb–7Zr–5Ta at temperatures ranging from 300 to 550 °C for different durations. They observed that over-aging occurred at all aging temperatures when the aging time exceeded 5 h, leading to a decrease in strength with the increase in aging time. Additionally, Hao et al. [12] identified a correlation between the elastic modulus and the volume fraction of precipitated α phase in the Ti–29Nb–13Ta–4.6Zr alloy. These findings underscore the significance of carefully controlling the aging parameters to achieve the desired mechanical properties.

Building upon prior work, our research team previously developed a novel metastable β-type Ti–25Nb–8Sn alloy [13], leveraging the biocompatible elements Nb and Sn. Ti–25Nb–8Sn exhibits a relatively low elastic modulus (~52 GPa), mitigating stress shielding effects, yet its strength (~962 MPa) requires improvement. This study aims to enhance the strength while maintaining the low elastic modulus of Ti–25Nb–8Sn through optimized aging heat treatment. By subjecting the alloy to 90% cold rolling followed by aging treatment, we seek to elucidate the effects of varying aging temperatures on phase precipitation and growth.

## 2. Materials and Methods

The Ti–25Nb–8Sn alloy (in mass%) utilized in this study was synthesized from a titanium sheet (99.9% purity), a niobium sheet (99.95% purity), and a tin wire (99.95% purity). The metal raw materials were placed in a concave copper crucible and melted and cast by using a commercial arc-melting vacuum-pressure-type casting system. To ensure homogeneous chemical composition, the alloy ingot was melted five times before being cast into a graphite mold during the sixth melting. The as-cast specimens were ground to a thickness of 9.4 mm by using sandpaper and cleaned in an ultrasonic bath with ethanol for 30 min. Subsequently, the specimen underwent repetitive cold rolling by using a rolling mill (5TSB; Jong Yih Co., Kaohsiung, Taiwan), with each rolling reducing the thickness by 0.4 mm until reaching a final thickness of 0.94 mm. The surface oxide layer of the rolled specimen was ground to a final level of 2000-grit paper and washed in ethanol by using an ultrasonic cleaner for 30 min. The rolled specimen was then placed in a quartz tube containing pure Ti as an oxygen getter material and heated in a preheated tubular furnace in a pure Ar atmosphere after several cycles of high-vacuum pumping. Aging treatments were conducted at temperatures of 300, 350, 400, 450, 500, 550, and 600 °C for 60 min, followed by air cooling to room temperature. The choice of 60 min aging time was based on previous findings that longer aging times (exceeding 5 h) can lead to over-aging effects, such as increased volume fraction of the α phase and consequent increase in the elastic modulus [11]. Additionally, higher aging temperatures accelerate over-aging [11]. To investigate the critical phase transformations and mechanical properties under varying aging temperatures, a shorter aging duration of 60 min was selected. This approach allows for a focused exploration of how aging temperature affects both the alloy’s microstructure and mechanical performance. The code names of the Ti–25Nb–8Sn specimens under various conditions are listed in Table 1.

Phase composition analysis of the specimens aged at various temperatures was conducted by using an X-ray diffractometer (D8 Advance; Bruker, Karlsruhe, Germany) operating at 40 kV and 40 mA in locked-coupled mode. The X-ray, excited by Cu-Kα (λ = 1.5405 Å), was filtered by a Ni filter. Scanning was performed in the range of 30–80° (2θ) with a scanning speed of 4°/min and a step scan increment of 0.02°. To accurately observe the diffraction peaks of the α and α″ phases, a slow scan was utilized for XRD analysis with a diffraction angle of 37–41° (2θ) and a scanning speed of 0.5°/min. Microstructural studies were carried out following standard metallographic procedures, which involved grinding with silicon carbide paper up to 2000 grit, polishing with colloidal silica-polishing suspension, and etching for 5 s in a solution composed of 4 mL of HF, 10 mL of HNO_3_, and 52 mL of H_2_O. Metallographic observation was conducted by using a metallographic microscope (Axio Lab. A1 Mat; Zeiss, Oberkochen, Germany) and a scanning electron microscope (SEM; S-4800; Hitachi, Tokyo, Japan).

The microhardness testing of the polished specimen was conducted by using a microhardness tester (HMV-20; Shimadzu, Kyoto, Japan) with a load of 0.98 N and a duration of 15 s. Ten separate measurements were taken from two specimens for each aging condition. Three-point bending tests were performed by using a desktop universal testing machine (HT-2102AP; Hung Ta Instrument, Taichung, Taiwan) at a crosshead speed of 0.5 mm/min, with a span length of 30 mm and a maximum deflection distance of 8 mm. The bending strength (σ) was determined by using the formula $\sigma =\frac{3PL}{2bh^{2}}$ [14], where P denotes the applied load (N), L is the span length (mm), b is the width of the specimen (mm), and h is the thickness of the specimen (mm). Furthermore, the elastic modulus (E) was calculated with the equation $E=\frac{PL^{3}}{4bh^{3}∆\delta }$ [14], where Δδ indicates the load displacement. The elastic admissible strain (EAS) was calculated by using the equation $EAS=\frac{\sigma_{y}}{E}$ [15], where σ_y_ represents the yield strength (MPa) and E denotes the elastic modulus (GPa). Given the expectation for biomedical implants to possess high yield strength and low elastic modulus, EAS serves as a suitable metric for evaluating the alloy for biomedical implant applications. Additionally, the elastic recovery angle was measured from changes in deflection angles before and after the release of the bending load. When the bending test reached the preset maximum deflection distance of 8 mm, denoted by θ_1_, the sample had its maximum deflection angle. Subsequently, upon removal of the load, the sample underwent elastic deformation, resulting in a deflection angle denoted by θ_2_. The elastic recovery angle of the alloy was determined by subtracting θ_2_ from θ_1_, with details reported in a previous study [14]. Fracture surfaces of broken samples were examined by using a scanning electron microscope (SEM; S-4800; Hitachi, Tokyo, Japan).

To investigate the corrosion properties of Ti–25Nb–8Sn samples under various conditions, experiments were conducted by using a potentiostat (PGSTAT12; Autolab, Utrecht, The Netherlands) in a phosphate-buffered saline (PBS) solution at 37 °C and pH 7.4. The experimental setup comprised the Ti–25Nb–8Sn sample as the working electrode, a silver/silver chloride (Ag/AgCl) electrode as the reference, and a platinum plate as the auxiliary electrode. The open circuit potential (OCP) for each sample was monitored for 60 min before commencing the potentiodynamic polarization tests, which involved scanning from −0.6 V to 1.6 V at a scan rate of 1 mV/s. Electrochemical impedance spectroscopy (EIS) was utilized to examine the passivation layer characteristics, applying a 10 mV voltage amplitude across a frequency range of 10^5^ to 10^−2^ Hz. Data from the experiments were processed and analyzed by using Metrohm Autolab NOVA 2.1 software. The PBS solution used for these tests was composed of 8.0 g/L NaCl, 0.2 g/L KCl, 1.44 g/L Na_2_HPO_4_, and 0.24 g/L KH_2_PO_4_.

## 3. Results and Discussion

### 3.1. Phase Identification

**Figure 1:**
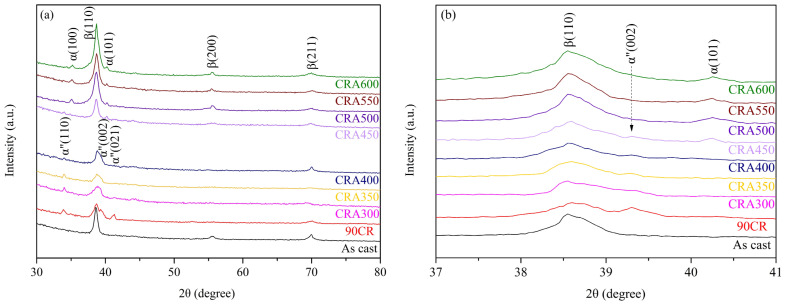
XRD diffraction patterns of Ti–25Nb–8Sn under various conditions. (**a**) Scanning speed of 4°/min in the diffraction angle of 30–80° (2θ) and (**b**) scanning speed of 0.5°/min in the diffraction angle of 37–41° (2θ).

Figure 1a displays the XRD diffraction patterns of Ti–25Nb–8Sn under various conditions. The patterns indicate that as-cast Ti–25Nb–8Sn is primarily in the β phase, while an α″ phase emerges after a 90% reduction in thickness through cold rolling. During aging heat treatment at different temperatures, the α″ phase gradually reverts to the β phase as the aging temperature increases. Table 2 summarizes the phases and phase volume fractions of the Ti–25Nb–8Sn specimens under various conditions, which are obtained from Figure 1a. Figure 1b presents the XRD patterns of Ti–25Nb–8Sn under various conditions, scanned at a slow speed of 0.5°/min within the angle range of 37–41°. The results show that as the aging temperature rises, the intensity of the α″-phase peak decreases while the β-phase diffraction peak intensity increases. At 450 °C, a weak α-phase diffraction peak begins to appear. When the aging temperature exceeds 500 °C, the α″ phase is completely converted into β phase, and a more pronounced α-phase diffraction peak is detected. This observation aligns with Plaine et al. [16], who noted that metastable β-type Ti alloys form stress-induced α″ phase after cold rolling, which then reverts to β phase after aging heat treatment.

Li et al. [17] investigated the aging treatment of the Ti–9.2Mo–2Fe alloy at 300 °C, 400 °C, and 500 °C for varying durations from 15 min to 72 h. They found that the isothermal ω phase (ω_iso_) appeared between 300 °C and 500 °C, with its quantity decreasing as the aging temperature or time increased. It was also suggested that at higher aging temperatures, the ω_iso_ phase could act as nucleation sites for the α phase, aiding its precipitation [18]. However, the XRD results (Figure 1) in this study do not show diffraction peaks of the ω_iso_ phase, possibly due to its nano-scale size or low content. The size and volume fraction of ω_iso_ particles depend on heat treatment temperature and duration [19]. The common size range of ω_iso_ phase formed during low-temperature aging (<500 °C) in Ti alloys is 2 to 100 nm [19]. Additionally, cold rolling can inhibit the formation of ω_iso_ phase due to the presence of numerous grain boundaries or dislocations, which prevent the collapse of the (111)β plane [20]. This renders the ω_iso_ phase challenging to detect. Inferred from the subsequent mechanical property results in this study, it is likely that ω_iso_-phase precipitations occur in the cold-rolled Ti–25Nb–8Sn alloy when aged between 300 °C and 450 °C. At temperatures above 500 °C, the ω_iso_ phase may transform into α phase, resulting in a distinct α-phase diffraction peak.

### 3.2. Microstructure

**Figure 2:**
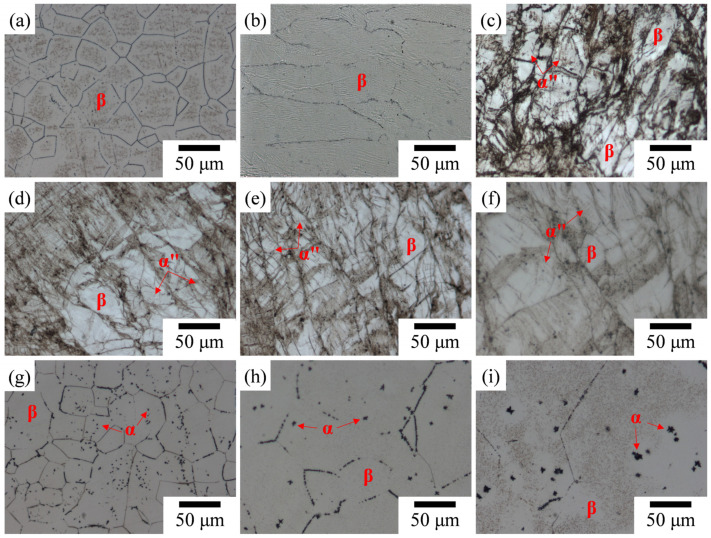
Optical micrographs of Ti–25Nb–8Sn under various conditions (direction of rolling from left to right). (**a**) As cast, (**b**) 90CR, (**c**) CRA300, (**d**) CRA350, (**e**) CRA400, (**f**) CRA450, (**g**) CRA500, (**h**) CRA550, and (**i**) CRA600.

The optical micrographs presented in Figure 2 depict the microstructure of Ti–25Nb–8Sn under various conditions. Initially, as-cast Ti–25Nb–8Sn reveals equiaxed β grains (Figure 2a), which undergo significant elongation following cold rolling (Figure 2b). Subsequent aging treatment at temperatures ranging from 300 °C to 450 °C results in the emergence of needle-like α″ phase alongside the β phase, as evident in Figure 2c–f. This observation aligns with previous findings by Wang et al. [21], who noted the formation of acicular α″ phase along the β-grain boundaries after similar cold rolling and aging heat treatment of Ti–30Nb–5Ta–6Zr. Additionally, Figure 2f highlights the precipitation of α-phase particles within the β grains at 450 °C. Notably, the fraction of the needle-like α″ phase diminishes in the alloy aged at 450 °C compared with temperatures between 300 °C and 400 °C, which is attributed to the partial reverse transformation of the α″ phase back to β phase. Following aging heat treatment at 500 °C or higher, the α″ phase completely reverts to β phase, disappearing entirely, while a substantial amount of granular α phase precipitates from both the grain boundaries and the matrix of the β phase. Moreover, the α phases formed after low-temperature aging exhibit a nano-scaled morphology (Figure 2f,g), with the particle sizes gradually increasing at higher aging temperatures (Figure 2h,i).

### 3.3. Mechanical Properties

#### 3.3.1. Microhardness

**Figure 3:**
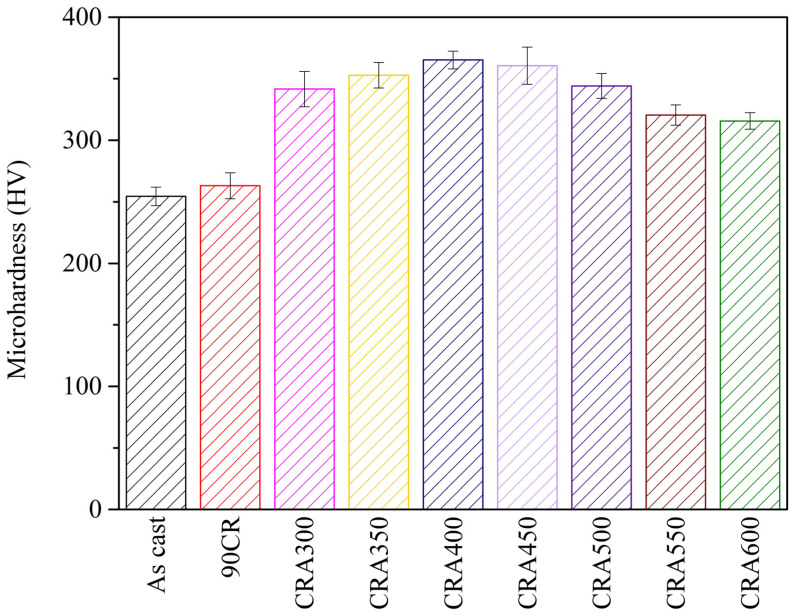
Microhardness of Ti–25Nb–8Sn under various conditions.

Figure 3 illustrates the microhardness values of Ti–25Nb–8Sn under various conditions. The as-cast specimen exhibits the lowest microhardness (253 HV), while a slight increase is observed in the cold-rolled specimen (90CR) with a microhardness of 263 HV. This modest increase can be attributed to the enhanced grain boundary and dislocation density induced by cold rolling. However, the presence of the cold rolling-induced α″ phase partially offsets this effect, resulting in the 90CR sample exhibiting only a marginal increase in hardness compared with the as-cast sample.

Upon aging treatment, the microhardness values of the specimens (ranging from 315 to 365 HV) significantly surpass those of both the as-cast and cold-rolled samples. This notable increase is attributed to precipitation strengthening facilitated by the α phase. With the increase in aging temperature, more α phase precipitates, consequently increasing the microhardness values. However, a peak microhardness of 365 HV is observed at the 400 °C aging temperature. Subsequent increases in aging temperature result in a gradual decline in microhardness, which is attributed to the coarsening of β and α grains.

#### 3.3.2. Bending Properties

**Figure 4:**
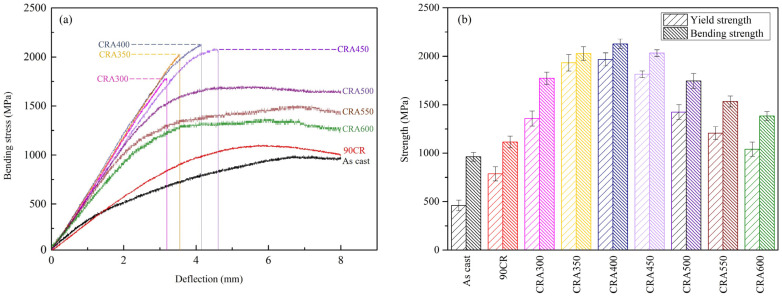
Bending properties of Ti–25Nb–8Sn from three-point bending test under various condition. (**a**) Stress–deflection profiles and (**b**) bending strengths and yield strengths.

Figure 4a depicts the bending stress–deflection profiles of Ti–25Nb–8Sn under various conditions. Notably, samples aged at temperatures ranging from 300 °C to 450 °C experience premature fracture before reaching the preset maximum deflection of 8 mm. The average fracture deflection increases with the aging temperature, with values of approximately 3.19 mm, 3.54 mm, 4.14 mm, and 4.61 mm being observed at 300 °C, 350 °C, 400 °C, and 450 °C, respectively. This trend suggests an association between fracture deflection and aging temperature, indicative of ω-phase precipitation-induced premature fracture within this temperature range. Remarkably, samples aged between 400 °C and 450 °C exhibit post-yield point fracture behavior, indicating a transition from brittle to ductile fracture behavior as the aging temperature increases, which is likely attributed to the gradual transformation of the ω phase into α phase [19].

Figure 4b presents the bending strength and yield strength in bending for Ti–25Nb–8Sn under various conditions. The aging temperature shows similar trends in affecting both the strength and microhardness of the alloy. The strengths of aging-treated samples are significantly higher than those of the as-cast and 90CR samples. As the aging temperature increases, so does the strength. At 400 °C, the alloy exhibits the highest bending and yield strengths (2127 MPa and 1960 MPa, respectively), which can be attributed to the effect of ω precipitation hardening. When the aging temperature exceeds 450 °C, strength values decrease as the temperature increases. Drawing from the experimental results presented in this section and the preceding sections on phase structure and microstructure, it can be inferred that temperatures exceeding 450 °C lead to the gradual dissolution of the ω_iso_ phase, which then acts as nucleation sites for the precipitation of the α phase. Since the hardness and strength of the α phase are lower than those of the ω phase, both the hardness and strength of the alloy decrease as the aging temperature increases. Similar observations were reported in the aging heat treatment of Ti–7Nb–10Mo, where the highest hardness value (535 HV) was achieved after aging at 400 °C. Conversely, increasing the temperature above 500 °C resulted in a gradual decrease in hardness values due to α-phase precipitation, with the lowest hardness value (401 HV) recorded at 650 °C [22].

#### 3.3.3. Elastic Properties

**Figure 5:**
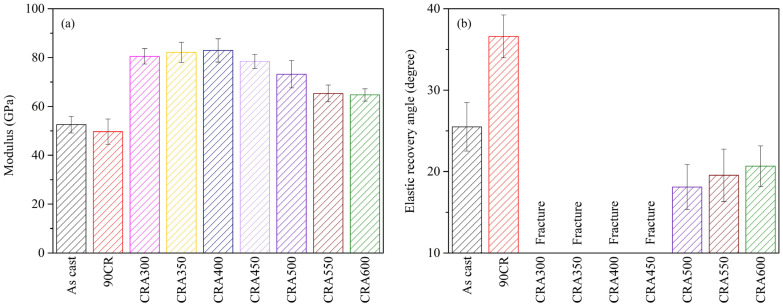
Elastic properties of Ti–25Nb–8Sn from three-point bending test under various condition. (**a**) Elastic modulus and (**b**) Elastic recovery angles.

The elastic moduli of Ti–25Nb–8Sn under various conditions are depicted in Figure 5a. The results reveal that the 90CR sample exhibits the lowest elastic modulus (49 GPa), primarily attributed to the formation of the stress-induced α″ phase. Furthermore, the decrease in elastic modulus may be related to texture evolution induced by severe cold rolling. Tane et al. found that the <100> orientation in Ti–29Nb–13Ta–4.6Zr alloy exhibits the lowest elastic modulus among all crystallographic orientations [23]. Conversely, the elastic moduli of the aging-treated samples (ranging from 64 to 82 GPa) at different temperatures surpass those of the as-cast (52 GPa) and 90CR specimens. Within the temperature range of 300–400 °C, the elastic modulus increases with the increase in aging temperature, peaking at 82 GPa at an aging temperature of 400 °C. This trend may be linked to the precipitation of the ω phase within the 300–400 °C range, a phenomenon corroborated by numerous studies [10,24]. Conversely, when temperatures exceed 400 °C, the elastic modulus gradually decreases, possibly due to the disappearance of the ω phase. Costa et al. [25] reported that the elastic modulus of Ti–30Nb–1Fe was 106 GPa after aging at 400 °C, reducing to 98 GPa at 500 °C, and further decreasing to 82 GPa at 550 °C, which is primarily attributed to the transformation from the ω phase to the α phase. Additionally, as α precipitation continues at higher temperatures, although the strength significantly diminishes, its effect on the elastic modulus is minor. Given that the elastic modulus is primarily governed by the constituent phases and elements of the alloy [1], the resulting elastic modulus values are quite similar when the aging temperature is 550 or 600 °C in this study.

Figure 5b illustrates the elastic recovery angles of Ti–25Nb–8Sn under various conditions. The elastic recovery angle of the 90CR sample, which contains β + α″ phases, is significantly higher than that of as-cast Ti–25Nb–8Sn with a single β phase. This is attributed to the superelastic behavior induced by the stress-induced α″ phase [26]. In the 90CR sample, more α″ phase is stress-induced during the three-point bending test, which reverses back to the β phase upon load release, enhancing the elastic recovery angle. Conversely, samples aged within the 350–400 °C range fracture during the three-point bending test, preventing the measurement of their elastic recovery angles. Among all the aged samples, the sample aged at 500 °C (CRA500) exhibits the lowest elastic recovery angle. As the aging treatment temperature increases, the elastic recovery angle of the samples gradually increases, which is attributed to the elimination of residual stresses.

#### 3.3.4. Fracture Surface Topography

**Figure 6:**
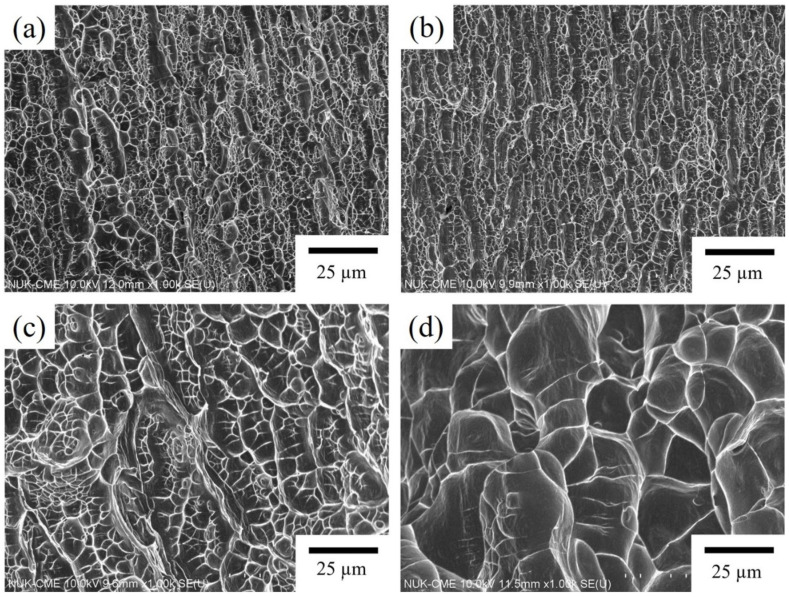
SEM images of the fracture surfaces of (**a**) CRA300, (**b**) CRA350, (**c**) CRA400, and (**d**) CRA450.

SEM images of the fracture surfaces of specimens aged at 300 °C, 350 °C, 400 °C, and 450 °C for 60 min following the bending test are presented in Figure 6. The images reveal the presence of dimples across all fracture surfaces. The dimples on the samples aged at 300 °C and 350 °C are fine (Figure 6a,b), whereas those on the samples aged at 400 °C and 450 °C are deeper and larger (Figure 6c,d). Azevedo et al. [27] reported similar features in Ti-Nb-Sn alloys subjected to aging heat treatment after cold rolling and solution treatment. They concluded that the diameter of the dimples correlates positively with the elongation of the alloy, with finer and shallower dimples indicating lower ductility. In alignment with this, the results of this study demonstrate that as the aging temperature increases from 300 °C to 450 °C, the dimples observed via SEM become progressively larger and deeper, suggesting an enhancement in the alloy’s ductility. After aging at 450 °C (Figure 6d), the presence of numerous large and deep dimples signifies enhanced ductility, consistent with the results depicted in Figure 4a.

#### 3.3.5. Elastic Admissible Strain (EAS)

**Figure 7:**
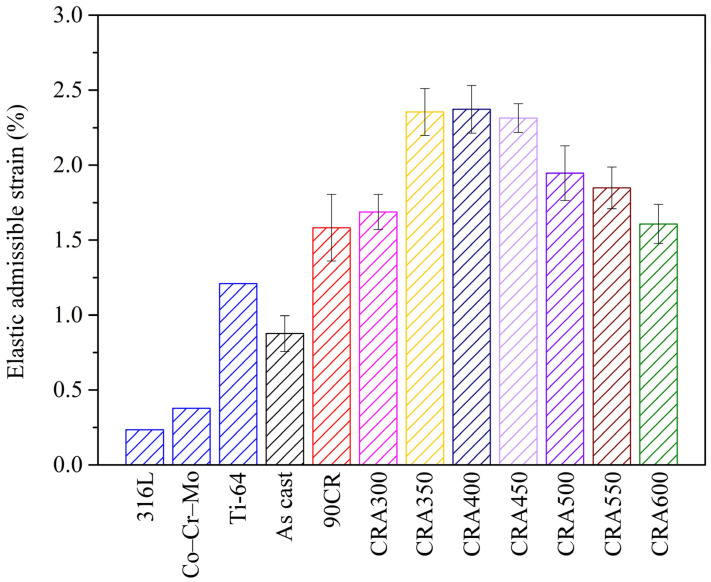
Elastic admissible strain of each Ti–25Nb–8Sn under various conditions, along with comparison alloys: 316L (316L stainless steel), Co–Cr–Mo, and Ti-64 (Ti–6Al–4V extra-low interstitials).

Figure 7 illustrates the EAS of Ti–25Nb–8Sn under various conditions, alongside data for 316L (316L stainless steel), Co–Cr–Mo, and Ti-64 (Ti–6Al–4V extra-low interstitials) [28] for comparative purposes. The elastic admissible strain, defined as the ratio of yield strength to elastic modulus, is a crucial mechanical parameter for evaluating implant materials [15]. For orthopedic implant applications, it is desirable to have a lower elastic modulus to reduce stress shielding and a higher yield strength to withstand loads. Therefore, maximizing the elastic admissible strain is advantageous.

The results in Figure 7 reveal that the CRA350, CRA400, and CRA450 samples exhibit higher EAS ratios due to the significant increase in alloy strength caused by ω precipitates. However, these samples are unsuitable for biomedical implants due to their relatively high elastic modulus and brittle characteristics. Among the ductile alloys studied (aged above 500 °C), the alloy aged at 550 °C shows a relatively high EAS ratio (1.93%) and a lower elastic modulus (65 GPa), significantly higher than the ratio (0.93%) of the as-cast Ti–25Nb–8Sn. Notably, the EAS ratio for the alloy aged at 550 °C is substantially higher than those of 316L (0.23%), Co–Cr–Mo (0.38%), and Ti-64 (1.21%), commonly used in clinical practice. These findings suggest that Ti–25Nb–8Sn aged at 550 °C for 60 min post-cold rolling holds potential as a biomedical implant material. The subsequent analysis focuses on the corrosion resistance of CRA550, with the as-cast and 90CR samples serving as control groups.

### 3.4. Corrosion Properties

#### 3.4.1. Potentiodynamic Polarization Tests

**Figure 8:**
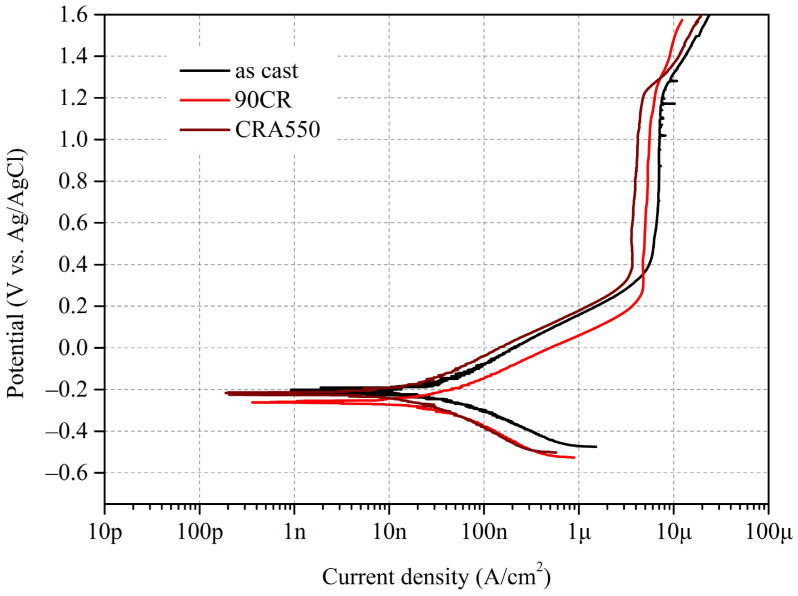
Polarization curves of as-cast Ti–25Nb–8Sn, 90CR, and CRA550 in a PBS solution at 37 °C.

The polarization curves of as-cast Ti–25Nb–8Sn (As cast), 90CR, and CRA550 in a PBS solution at 37 °C are displayed in Figure 8. Table 3 summarizes the electrochemical parameters obtained from potentiodynamic polarization tests for these samples in PBS solution at 37 °C. These parameters include corrosion potential (E_corr_), corrosion current density (i_corr_), passivation potential (E_pass_), passive current density (i_pass_), anodic slope (β_a_), cathodic slope (β_c_), polarization resistance (R_p_), and corrosion rate.

The E_corr_ values for the as-cast and CRA550 samples are slightly higher than that for 90CR, which is attributed to the introduction of residual stresses and lattice distortions from cold rolling, making the latter more susceptible to corrosion. Additionally, the i_pass_ values for 90CR and CRA550 are significantly lower than that of the as-cast sample. The high density of lattice defects and refined grains produced by the cold rolling process enhances the corrosion resistance of the alloy [29], resulting in a lower i_pass_ value for 90CR. Furthermore, aging treatment further reduces surface defects and releases residual stresses, promoting a more uniform distribution of precipitates and stabilizing the grain structure; thus, CRA550 exhibits the lowest i_pass_ value.

Regarding passivation capability, the as-cast sample exhibits metastable pitting at high potentials (>1.0 V), characterized by the rapid reformation of the surface oxide passive layer after it is disrupted. Additionally, no pitting was observed for 90CR and CRA550 even when the test potential was increased to 1.6 V. The 90CR sample has the lowest E_pass_ value, indicating that it can quickly form a passive layer upon corrosion due to grain refinement from cold rolling, which provides more nucleation sites for the oxide passive layer [30]. Furthermore, the R_p_ value for CRA550 is significantly higher than those for 90CR and the as-cast sample, and CRA550 also exhibits the lowest corrosion rate.

#### 3.4.2. Electrochemical Impedance Spectroscopy (EIS) Test

**Figure 9:**
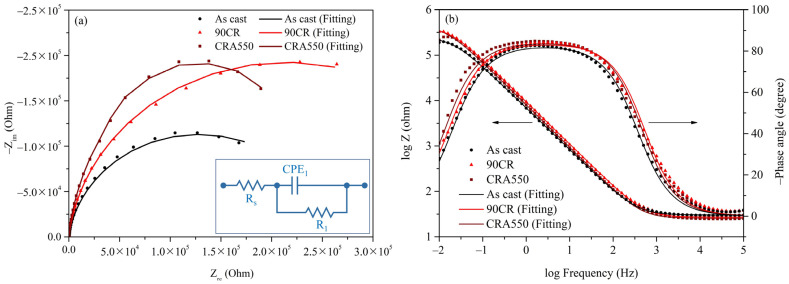
EIS results for as-cast Ti–25Nb–8Sn, 90CR, and CRA550 in a PBS solution at 37 °C with solid lines representing results simulated by Metrohm Autolab NOVA 2.1. (**a**) Nyquist plot and (**b**) Bode plot. The insert in (**a**) is the equivalent electrical circuit model utilized for fitting the EIS data.

The EIS results for as-cast Ti–25Nb–8Sn (As cast), 90CR, and CRA550 in a PBS solution at 37 °C are depicted in Figure 9. Nyquist and Bode plots are presented in Figure 9a and Figure 9b, respectively. The inset in Figure 9a displays the equivalent electrical circuit (EEC) model used to fit the EIS data, with solid lines indicating the simulated results produced by Metrohm Autolab NOVA 2.1. In this model, R_s_ represents electrolyte resistance, CPE_1_ is the constant phase element of the passivation layer, and R_1_ is the polarization resistance of the passivation layer. The EIS data for each sample, including parameters such as R_s_, CPE_1_, R_1_, the deviation parameter for CPE_1_ (n_1_), effective constant capacitance (C_eff_), and chi-square value (χ^2^), were fitted by using the EEC model shown in the inset of Figure 9a with Metrohm Autolab NOVA 2.1 software and are summarized in Table 4. The formula for calculating C_eff_ is as follows [31]: $C_{eff,1}={{CPE}_{1}}^{\frac{1}{n_{1}}}{R_{1}}^{\frac{1-n_{1}}{n_{1}}}$. The χ^2^ values for each sample were around 10^−4^, indicating the accuracy of the equivalent circuit fitting.

The Nyquist capacitive semicircle diameters of 90CR and CRA550 are significantly larger than that of the as-cast sample (Figure 9a), indicating superior charge transfer resistance. Additionally, the R_1_ values of 90CR and CRA550 are noticeably higher than that of the as-cast sample (Table 3), reflecting higher stability of their passive oxide layers. Moreover, the CPE_1_ values for 90CR and CRA550 are lower than that of the as-cast sample, suggesting a less defective structure of the passive oxide layer [32]. In the Bode plot (Figure 9b), at the terminal frequency (10^−2^ Hz), CRA550 exhibits the highest impedance, further confirming the high stability of its passive film. As explained in the previous section, the refined grains of 90CR after cold rolling provide more nucleation sites for the passive oxide layer, resulting in a thicker and more complete passive layer. Furthermore, the heat treatment process for CRA550 significantly reduces the negative impact of surface defects and residual stresses, leading to a denser and more uniform passive oxide layer. The combined results of potentiodynamic polarization and EIS indicate that CRA550 demonstrates the best corrosion resistance.

## 4. Conclusions

This study investigated the phase transformations of Ti–25Nb–8Sn alloy after 90% cold rolling followed by aging treatment at various temperatures for 60 min and their impact on mechanical properties. Aging at temperatures between 300 °C and 450 °C significantly increases the bending strength and elastic modulus of Ti–25Nb–8Sn alloys, exhibiting brittle fracture characteristics. This behavior is primarily attributed to the precipitation of the ω phase within this temperature range. However, at higher aging temperatures (above 450 °C), the α″ phase predominantly transforms back to β phase, and the ω phase converts into α phase. This phase transformation results in reduced elastic modulus and improved ductility of the alloy.

Therefore, the findings indicate that Ti–25Nb–8Sn subjected to 90% cold rolling and subsequent aging at 550 °C for 60 min demonstrates ductile properties, an optimal combination of mechanical properties, and excellent corrosion resistance. These characteristics suggest that the alloy has significant potential for use as a biomedical implant material.

## Figures and Tables

**Table 1:** The code names of Ti–25Nb–8Sn specimens under various conditions.

Code Name	Processing Condition
As cast	As cast
90CR	90% cold rolling
CRA300	90% cold rolling + aging at 300 °C for 60 min
CRA350	90% cold rolling + aging at 350 °C for 60 min
CRA400	90% cold rolling + aging at 400 °C for 60 min
CRA450	90% cold rolling + aging at 450 °C for 60 min
CRA500	90% cold rolling + aging at 500 °C for 60 min
CRA550	90% cold rolling + aging at 550 °C for 60 min
CRA600	90% cold rolling + aging at 600 °C for 60 min

**Table 2:** Phases and phase volume fractions of Ti–25Nb–8Sn specimens under various conditions.

Alloy	Phase Volume Fractions (%)		
	β	α″	α
As cast	100	0	0
90CR	56	44	0
CRA300	68	32	0
CRA350	70	30	0
CRA400	71	29	0
CRA450	90	5	5
CRA500	93	0	7
CRA550	93	0	7
CRA600	91	0	9

**Table 3:** Corrosion characteristics obtained from potentiodynamic polarization tests for as-cast Ti–25Nb–8Sn, 90CR, and CRA550, including corrosion potential (E_corr_), corrosion current density (i_corr_), passivation potential (E_pass_), passive current density (i_pass_), anodic curve slope (β_a_), cathodic curve slope (β_c_), polarization resistance (R_p_), and corrosion rate.

Alloy Conditions	E_corr_ (V)	i_corr_ (A/cm^2^)	E_pass_ (V)	i_pass_ (A/cm^2^)	β_a_ (V/dec)	β_c_ (V/dec)	R_p_ (MΩ·cm^2^)	Corrosion Rate (mm/year)
As cast	−0.21	1.08 × 10^−8^	0.36	4.85 × 10^−6^	0.10	0.07	1.66	8.72 × 10^−5^
90CR	−0.25	7.50 × 10^−9^	0.22	3.94 × 10^−6^	0.06	0.07	1.87	6.07 × 10^−5^
CRA550	−0.21	3.77 × 10^−9^	0.33	3.23 × 10^−6^	0.06	0.06	3.46	4.39 × 10^−5^

**Table 4:** Electrochemical impedance spectroscopy (EIS) data for as-cast Ti–25Nb–8Sn, 90CR, and CRA550, featuring electrolyte resistance (R_s_), constant phase element of the passivation layer (CPE_1_), polarization resistance of the passivation layer (R_1_), deviation parameter for CPE_1_ (n_1_), effective constant capacitance value (C_eff_), and chi-square value (χ^2^). Data were fitted based on the equivalent electrical circuit (EEC) model in Figure 9a by using Metrohm Autolab NOVA 2.1 software.

Alloy Conditions	R_s_ (Ω·cm^2^)	CPE_1_ (10^−5^ F·cm^2^)	R_1_ (kΩ·cm^2^)	n_1_	C_eff_ (10^−5^ F·cm^2^)	χ^2^ (10^−4^)
As cast	30.3	26.7	256	0.920	3.145	1.2022
90CR	27.3	19.2	429	0.931	2.256	1.4815
CRA550	25.6	22.5	386	0.935	2.639	5.4953

## Data Availability

Data are contained within the article.
